# Role of bimaxillary surgery in treatment of high respiratory obstructive sleep apnea syndrome

**DOI:** 10.1186/1746-160X-10-S1-O9

**Published:** 2014-12-12

**Authors:** Ashraf MS Ghanem

**Affiliations:** 1 Department of Oral and Maxillofacial Surgery, Faculty of Dentistry, Minia University, Minya, Egypt

## 

High respiratory obstructive sleep apnea syndrome (OSAS) is a potentially disabling condition, characterized by excessive daytime sleepiness, disruptive snoring, recurrent episodes of apnea (or hypopnea) and nocturnal hypoxemia. It is a sign of increased pharyngeal airflow resistance (apnea hypopnea index (AHI ≥ 5).

Twelve patients suffering from mild-to-severe OSAS received CPAP treatment and the ANB angle of their cephalometric analysis is < 1 degree and therefore, diagnosed as a hidden maxillo-mandibular skeletal deformity.

In this study, an innovative technique is designed to modify the maxillary mandibular advancement (MMA) surgical principle method and applied to treat these cases. While tracheal, nasal and uvulo-palatal tissues were not collapsing and obstructing the upper airway region, skeletal advancements was used to create and enlarge tongue room space resulting a patent posterior airway space (PAS). Digitally measured diameter at the epiglottis region in oro-pharyngeal airway space, was implemented, assuming a cylindrical model, to determine the total air volume change.

Results showed a marked positive air flow gain (approximately 1.1 ± 0.2 cm^3^; 12 months postoperative) with statistical significant differences (p < 0.01). Thus it has been concluded that MMA is very useful in managing severe class III skeletal deformity having OSAS via increasing the total air column volume gain in the upper oro-pharyngeal airway space at the epiglottis region. It may, therefore, be advocated that it could be considered as a standard surgical treatment after failure of CPAP existed.

